# Policy action points and approaches to promote fertility care in The Gambia: Findings from a mixed-methods study

**DOI:** 10.1371/journal.pone.0301700

**Published:** 2024-05-14

**Authors:** Anna Afferri, Susan Dierickx, Mustapha Bittaye, Musa Marena, Allan Antony Pacey, Julie Balen

**Affiliations:** 1 School of Health and Related Research–ScHARR, The University of Sheffield, Sheffield, United Kingdom; 2 Institute of Tropical Medicine, Antwerp, Belgium; 3 Department of Clinical Sciences, Research Centre Gender, Diversity and Intersectionality—RHEA, Vrije Universiteit Brussel, Brussel, Belgium; 4 Ministry of Health, Banjul, The Gambia; 5 School of Medicine and Allied Health Sciences, University of The Gambia, Banjul, The Gambia; 6 School of Medical Sciences, Faculty of Biology, Medicine and Health, University of Manchester, Manchester, United Kingdom; 7 School of Allied and Public Health Professions, Canterbury Christ Church University, Canterbury, United Kingdom; 8 Medical Research Council–MRC Unit The Gambia at LSHTM, Fajara, The Gambia; The University of Texas Health Science Center at Houston, UNITED STATES

## Abstract

**Introduction:**

In the Global South, (in)fertility care is scarcely recognized as a priority, yet the government of The Gambia has recently included it as one of the key priorities in its reproductive health strategic plan. This inclusion appears to be the result of years of engagement between policy actors, academic researchers, and activists in the field of reproductive health and specifically of infertility. However, the operationalization of the strategic plan may be hampered by multiple factors. The research aims to identify and analyze challenges that may impede the effective implementation of the strategic plan, thereby providing policy action points and practical guidance into the operationalization of (in)fertility care in the context of The Gambia’s health system.

**Methods:**

This is a mixed-methods study with data from a survey and semi-structured interviews collected between 2020 and 2021 in The Gambia that were separately published. In this paper, we present the triangulation of quantitative and qualitative data using a convergence coding matrix to identify relevant policy action points.

**Results:**

Six fertility care policy action points, driven by data, arose from the triangulation and interpretation process, specifically: (i) establishing and maintaining political commitment and national priority for fertility care; (ii) creating awareness and increasing the involvement of men in SRH and fertility; (iii) ensuring data-driven health policymaking; (iv) offering and regulating affordable IVF alternatives; (v) improving knowledge of and means for fertility care provision; and (vi) enhancing the collaboration among stakeholders and building links with the private healthcare sector.

**Conclusion:**

This study found the implementation of the fertility care-related activities in the reproductive health strategic plan may face challenges that require careful mitigation through a holistic approach. Such an approach conceptualizes infertility not just as a biomedical issue but as a broader one that incorporates educational and socio-emotional aspects, including male and (not only) female involvement in sexual and reproductive health. Moreover, it is supported by a comprehensive health management information system that includes capturing data on the demand for, and access to, infertility services in The Gambia health system.

## Introduction

Fertility care is defined as a package of “interventions that include fertility awareness, support, and management with an intention to assist individuals and couples to realize their desires associated with reproduction and/or to build a family” [1]. In both high-income and low- and middle-income countries (LMICs), health systems do not always provide a comprehensive package of fertility care, while in LMICs very few components of the full package are available [2]. In general, national health systems are more inclined towards delivering infertility screening and diagnostics services, with the private sector tending to fill in the treatment gaps [3]. Moreover, in some LMICs, fertility care is scarcely recognized as a health issue, and even when included in national reproductive health policies, the implementation phase is frequently hampered by multiple health systems challenges such as poor governance and leadership, scares financial resources, and untrained health providers [4].

The failure to include and/or implement fertility care in national health programs is heavily influenced by fertility rates, especially in LMICs, as well as concerns about overpopulation and the high cost of assisted reproductive technologies (ART) [5].

Local governments in LMICs choose to focus their resources on lowering fertility rates rather than addressing involuntary infertility (the inability to become pregnant when desired), and international development agencies frequently underestimate the need for fertility care, meaning that there are few resources to draw on [6]. Yet, several LMICs have indeed engaged with fertility care in their national health policies [7–9]. This is the case, among other, for Uganda, Ethiopia, Iran, Morocco, and Thailand [10–13]. Morocco and Thailand, for example, both strengthened their national reproductive health policies and infertility services, and fertility care was incorporated as an ‘*essential*’ intervention in the Thai National Reproductive Health Strategy in 2020 [14].

When delivered in contexts of the Global South, mainly by the private sector, safety standards and ethical principles might be not enforced, and public health institutions may have little to no authority in regulating the private provision of fertility care [9, 15]. However, supporting sexual and reproductive rights that include fertility care is one of many compelling public health arguments to strengthen health systems. Yet, there are gaps in the understanding of how healthcare sectors (public and private) manage fertility care, most significantly during its implementation phase.

In The Gambia, West Africa, the government set priorities for fertility care in its new reproductive health strategic plan for 2022–2026 [16]. In order to understand potential challenges and opportunities that would arise during the implementation of the strategic plan, we conducted a mixed-method study on fertility care processes in the Gambian health system. Here we present the outcomes from the triangulation and interpretation of the quantitative and qualitative components of the study which generated policy action points aiming to support the implementation of the reproductive health strategic plan, and specifically the key priority that targets infertility management.

## Materials and methods

### Study settings

This study was carried out in The Gambia, West Africa, the smallest country within mainland Africa. The Gambia is surrounded by Senegal, with the exception of its Atlantic coast, and is divided into northern and southern areas by The Gambia River that runs through its center. Its population was estimated at 2.5 million inhabitants in 2022 [17]. The Gambia is divided into eight local government areas (LGAs), including the capital Banjul, with a further division of the LGAs into forty-three districts. All eight LGAs were included in the study sample, representing the country in its entirety. Considering the research was conducted mainly at health facility level, not all districts were selected in the sample.

### Study design and participant recruitment

We applied a mixed-methods design in which quantitative data from a cross-sectional survey [18] and qualitative data from semi-structured interviews [19] were triangulated and interpreted [20–22]. No a priori sample size calculation was done.

#### Quantitative data

The data collection for the survey started on March 2021 and concluded on August 2021. The aim of the survey was to assess the availability of infertility services in public and private facilities in The Gambia. Survey data were collected in-person from 38 health facilities (20 public; 18 private) purposely sampled to represent secondary and tertiary-level facilities distributed over the entire country. All public health facilities included in the secondary and tertiary-level of the health system were retained for the sample. Private facilities were selected through simple randomization from a pre-existent list. The results of this survey were published in 2022 [14], and can be summarized as follows: (i) basic screening of infertility was equally available in both public and private facilities; (ii) advanced infertility diagnostic and treatment services were mainly available in the private sector and far less so in the public sector; (iii) assisted reproductive technologies (ART) were not available in The Gambia; and (iv) two-third of the health facilities did not collect or report data on infertility.

#### Qualitative data

Qualitative data was collected via semi-structured interviews with a total of 49 policymakers, policy implementers, and health practitioners. Participants were selected for their direct expertise in health policy development and implementation, providing valuable insights into the operationalization of fertility care. Snowballing was applied at each interview [23]. The findings from the qualitative research were recently published [19]. The interviews were conducted with central and regional representatives from the Gambian Ministry of Health (MoH), a local NGO committed to addressing fertility awareness in The Gambia, and health practitioners working in the public and private health facilities that were sampled for the survey. In summary, the qualitative research identified several key challenges to the successful implementation of fertility care: (i) a lack of routinely collected infertility data; (ii) an absence of financial protection mechanisms for patients and/or a specific budget for infertility; (iii) limited cooperation between the public and private sectors in the provision of fertility care; and (iv) gaps in fertility care training among health practitioners.

### Data triangulation, analysis and interpretation

The triangulation process for this mixed-methods study began with the creation of a protocol (**S1 Table. Triangulation protocol**), and included actively looking for patterns (themes) that emerged by synthesizing and comparing within and across the quantitative and qualitative datasets. Themes were categorized by hand, based on conceptual similarities. Data from both the quantitative and qualitative datasets were then interpreted manually using a convergence coding matrix [24]. Findings were categorized as ‘convergent’ if they directly concurred, ‘complementary’ if they offered corresponding information on a similar matter, ‘silent’ if there was no overlap or connection between datasets, and ‘dissonant’ if the datasets contradicted one another. To develop cross-linkages within the themes, and provide practical recommendations, an additional column was added to the coding matrix and labeled ‘policy action points’. As a reminder, quantitative data were analysis with IBM SPSS version 26 and qualitative data with NVivo Pro 1.6.1 [2, 19].

### Ethics statement

This study was approved by The Gambia Government and Medical Research Council Gambia (MRCG) at the London School of Hygiene and Tropical Medicine Joint Ethics Committee (Reference 22446) and The University of Sheffield–School of Health and Related Research (ScHARR) Research Ethics Committee (Reference 03785–038109). Written informed consent was obtained from all participants prior to completing in the survey, and the confidentiality of the data was assured. All methods were performed in accordance with the relevant guidelines and regulations (Declaration of Helsinki).

## Results

Using as a guide the conceptual framework of enablers of fertility care policymakers [2], a total of thirteen themes were generated by comparing information from the two datasets (Table 1). Six out of 13 (46%) themes showed convergence of findings between the quantitative and qualitative datasets, while three out of 13 (23%) were complementary, and four out of 13 (31%) were silent. No dissonance of findings was identified. The lack of certain themes in one dataset but their presence in another (silence), might be attributed to the nature and method of data collection [24].

**Table 1:** Themes identified during the triangulation process, and level of convergence.

Themes	Level of convergence
**1. Perceived importance of infertility among policymakers**	Complementarity
**2. Mechanisms of funding fertility care**	Silence
**3. Attending infertility consultations**	Convergence
**4. Fertility awareness**	Silence
**5. Health information system**	Convergence
**6. Availability of ART**	Convergence
**7. Fertility care guidelines**	Silence
**8. Training on fertility care**	Convergence
**9. Infertility services availability**	Complementarity
**10. Integration of infertility within RH services**	Convergence
**11. Medicines and supplies for fertility care**	Convergence
**12. Collaboration among fertility care actors**	Silence
**13. Private care**	Complementarity

The four ‘silent’ themes, namely: (i) mechanisms for funding fertility care; (ii) fertility awareness; (iii) fertility care guidelines; and (iv) collaboration among fertility care actors, are equally important in the triangulation process because they show the power of one research method over another. Six policy action points emerged from the data triangulation that were linked to the above mentioned themes, namely: (i) establishing and maintaining political commitment and national priority for fertility care; (ii) creating awareness, and increasing the involvement of men in sexual and reproductive health (SRH) and fertility; (iii) ensuring data-driven health policymaking; (iv) offering affordable IVF alternatives and regulation of standards; (v) improving knowledge of and means for fertility care provision; and (vi) enhancing the collaboration among stakeholders and building links with the private healthcare sector (Table 2). Each policy action point is discussed in detail below and was identified merging current literature with data triangulation.

**Table 2:** Convergence coding matrix and policy action points.

*Themes*	*Cross-sectional survey (quant)*	*Semi-structured interviews (qual)*	*Convergence assessment*	*Policy Action Points*
**Perceived importance of infertility among policymakers**	66% of health facilities cited low priority for infertility and fertility care matters among policymakers.	Both health providers and policymakers/ implementers were aware of infertility as a medical and social issue in The Gambia. However, infertility is not perceived as important when compared to other health conditions.	*Complementarity*: other health priorities in The Gambia were seen as more important than infertility. A strategic plan to implement fertility care is needed.	Establishing and maintaining political commitment and national prioritization for fertility care
**Mechanisms of funding fertility care**		The allocation of finances to support fertility care was noted as a serious concern among policymakers and health providers. At the time of data collection, The Ministry of Health had not yet dedicated a budget for the implementation of fertility care. External funding from international development partners or the private sector was not available for fertility care interventions. The health insurance scheme was mentioned as a potential way to decrease out-of-pocket expenditure for fertility care.	*Silence*: funding mechanisms not questioned in the quantitative investigation.	
**Attending infertility consultations**	82% of the facilities reported that both members of the couple *never* or *occasionally* attend initial infertility visit together; while only 58% of the facilities reported that both members of the couple *often* or *usually* attend follow-up visits together.	Participants acknowledged that the limited involvement of men in the investigation and treatment of infertility-related issues could hamper successful treatment options for both members of the couple.	*Convergence*: in The Gambia, men’s participation in the diagnostic and curative journey for infertility is very limited.	Creating awareness and increasing the involvement of men in SRH and fertility
**Fertility awareness**		Participants had little recollection of any fertility awareness activity promoted by the government or any other institution, with a few exceptions of radio and TV talks. No specific fertility awareness plan was implemented or available.	*Silence*: fertility awareness not discussed in the quantitative investigation.	
**Health information system**	74% of the health providers did not collect data on infertility or did not know if data was collected and/or communicated to the MoH.	Participants’ views on infertility data collection and reporting confirmed that consultations for infertility were not captured nor reported due to an absence of infertility focus on the collection forms. If reported, data on infertility were said to be aggregated with other conditions. The MoH did not formally request data concerning infertility.	*Convergence*: data collection form lacking a dedicated space to capture infertility data. Infertility data not requested by the MoH. When reported, data on infertility were said to be merged with other health conditions.	Ensuring data-driven health policymaking
**Availability of ART**	100% of the health facilities reported unavailability of ART.	Participants cited that ART are not available in the country.	*Convergence*: ART not currently available in The Gambia.	Offering affordable IVF alternatives and regulation of standards
**Fertility care guidelines**	82% of facilities noted that not having a national guidance concerning fertility care is a barrier to full integration of services into existing reproductive health programs.		*Silence*: issue not explored in qualitative research.	
**Training on fertility care**	84% of the facilities cited that a lack of specialized fertility care training for health providers impacts the full integration of infertility services.	Participants reported not being fully trained in infertility management and specifically in ART. The little information they had on infertility was received in nursing schools or universities, and/or during on-job training. A few medical doctors beneficed of a scholarship from a private foundation to be train abroad on IVF.	*Convergence*: fertility care and infertility management training is scarce.	Improving knowledge of and means for fertility care provision
**Infertility services availability**	66% of the health facilities declared infertility services availability. 65% of public facilities and 67% of private clinics offer screening and diagnostic services for infertility. Treatments for infertility is mostly available in private clinics.	Respondents recognized that fertility care provided by private clinics has a wider range of available investigations and treatments compared with public facilities. Informal health system often used as primary entry point for seek care for infertility.	*Complementarity*: basic investigations for infertility are available in the public sector but majority of technologically advanced diagnostic and treatment services are delivered by the private sector.	
**Integration of infertility within RH services**	88% of the facilities providing infertility services have integrated them into existing reproductive health services, mainly within gynecology and family planning clinics. Three *for-profit* private clinics provide a standalone service dedicated to fertility care.	Most public facilities that provide infertility services have managed to integrate them in the current delivery of reproductive health services. These services were mostly delivered in family planning or gynecology clinics. In private clinics, infertility services often standalone.	*Convergence*: health facilities providing infertility services managed to integrate them into current reproductive health programs. In the private sector, fertility care is provided as standalone service.	
**Medicines and supplies for fertility care**	79% of the health facilities reported shortage of equipment, supply, and 74% shortage of medication as a barrier for infertility services provision. 65% of the public health facilities and 67% of the private clinics reported availability of screening and diagnostic services for infertility. Treatment for infertility is mostly available in the private sector.	Participants shared concerns about the ability of the MoH to fully commit to and support infertility-related activities due to shortage in medicines and unavailability of equipment dedicated to infertility investigations and treatment.	*Convergence*: medicines and technology for fertility care not systematically available in the public health facilities. More availability of infertility services is cited in the private care.	
**Collaboration among fertility care actors**		Participants from health facilities confirm little interaction with the MoH regarding fertility care directives. Collaboration between public-private, and informal sectors is scant as well as with UN agencies and international development partners.	*Silence*: Collaboration not discussed in the quantitative investigation, however reported as missing in different levels of interaction.	Enhancing collaboration among stakeholders and building links with private healthcare sector
**Private care**	67% of the clinics with available infertility services were private.	Comprehensive census of private clinics operating in the country is missing, but it is reported that most infertility services are available in the private sector. Cost of these services is one of the main causes impeding access to fertility care.	*Complementarity*: the private sector is the most comprehensive provider of fertility care in The Gambia. The health system lacks an updated census of the private clinics delivering fertility care.	

## Discussion

### Establishing and maintaining political commitment and national priority for fertility care

According to Fox et al. (2015), the extent of political commitment can vary from: (i) ‘expressed commitment’ or verbal support on a health issue by policymakers and leadership; (ii) ‘institutional commitment’ or the creation of policies and national guidelines to support and implement a health issue; and (iii) ‘budgetary commitment’ or the allocation of a dedicated budget to a specific health issue. The expression of engagement, without policy, action plan, and budgetary apportionment, is not a credible commitment and is seen as ‘rhetorical’ [25]. The engagement of The Gambia government toward fertility care appears very much aligned with the institutional commitment perspective because of the recent creation (Dec 2022) of a specific and dedicated strategic plan to challenge infertility as part of a broader strategy on SRH.

However, a strong political commitment toward a specific health matter is not always the sole driver favoring prioritization and policy creation and/or reform. Other factors exist that can have equal or even stronger influence forming political commitment, including social and religious values, activism, and the desire of politicians to stay in power [26]. Those drivers may work with or against political will and can influence policymaking and policy reform. Fertility care interest could follow a similar trajectory, and in the case of The Gambia, the involvement, coordination, and collaboration of the multiple national and international stakeholders working to enhance SRH outcomes could generate (and maintain) interest in infertility and push forward for a more holistic implementation of fertility care.

### Strategic framing

How health concerns are perceived and understood may be influenced by strategic framing. Framing entails using a multitude of analogies or arguments to draw the audience’s attention to certain parts of a subject to elicit a positive response [27, 28]. Through open and inclusive dialogue, views are challenged and changed [29]. Strategic framing can be applied to bring SRHR concerns (including infertility) to the attention of the government and international agendas and to influence decision-making processes. For infertility, commitment, and priority could be achieved by influencing health policy audiences and involving stakeholders through conducting health research on fertility care, among others. When health research addresses national priorities and action points with consideration of the context (such as funding and service delivery), they are more likely to be adopted at the national level [30, 31]. The creation in 2021 of the Fertility Care Network in the Global South (www.fertilitycareforall.org) whose members are mainly academics, but also policymakers has have a positive impact. Gambian policymakers who are within this network have been involved in the research process allowing to sustain fertility care prioritization. This has resulted in the creation of key interventions for infertility embedded in the new reproductive health strategic plan 2022–2026.

### Financial engagement

A promising body of knowledge has explored the links between social development derived from public financing of ART with the economic benefits generated by the return on investment for each child born via IVF [32, 33]. This was further supported by studies in which infertility insurance schemes were assessed for having effects on female infertility prevalence. Particularly, when infertility is covered by insurance schemes a reduction in female infertility is noted and women with insurance coverage had a higher likelihood of undergoing several IVF treatments, raising their cumulative birth rate [34]. The Gambia has demonstrated its commitment to upholding universal health coverage (UHC) standards by passing “The National Health Insurance Bill—2021” and by preparing to launch a National Health Insurance Scheme (NHIS). Additionally, the Ministry of Health pledged that both public and private health facilities will have access to the NHIS. This is important because previous research has evidenced that the majority of infertility treatments are delivered by the private sector [18]. However, there are also potential challenges in financing fertility care in The Gambia, such as continuity of the national budget and/or the viability of funding to maintain fertility care. In low-income nations like The Gambia, where public resources committed to health are already stretched, expenditures (partial or entire) for fertility care may negatively impact the long-term sustainability of services. This is much more evident in African countries where costs associated with infertility treatment and the Gross Domestic Product (GDP) is negatively linked, with fertility care costs reaching up to 200% of GDP per capita [35].

### Creating awareness and increasing the involvement of men in SRH and fertility

People with infertility fear discrimination and stigma, and this is one of the main reasons for keeping their status hidden [36]. In The Gambia one exception is represented by the *kanyalengs*, a group of women who publicly and explicitly share their childless status [37–39]. Members of these groups shared reproductive setbacks through dances, rituals, and prayers, and help women socially, emotionally, and occasionally financially [39]. In the country, Safe Haven Foundation (SHF - www.facebook.com/foundationsafehaven/"www.facebook.com/foundationsafehaven/) operates. Safe Haven Foundation is a local non-governmental organization that supports women and men through their fertility journey focusing on providing psychological help to deal with infertility, miscarriages, and other fertility issues. *Dimbayaa* is an organization of health professionals from the Netherlands who aim to bring attention toward the problem of infertility in Sub-Saharan Africa. The presence of both these organizations and the *kanyalengs* is of great importance for men and women living with infertility in the country and offers a window of opportunity to target fertility awareness starting from the community level. However, the data triangulation shows a scarce recollection of any fertility awareness-related activities, and there is a need, for the MoH, to include these organizations more holistically in the discussions about fertility care.

Another element that emerged from the triangulation of the datasets, regards the involvement and participation of men in fertility care. In general terms, male participation in SRH has historically suffered from conceptual uncertainty, and the emergence of the slogans such as ‘men as clients, men as partners, and men as agents of social change’ brought a different perspective on men’s involvement in the reproductive health matters [40, 41]. Men as Partner (MAP) is an approach that could effectively address the involvement of men in fertility care because it is a multidimensional intervention intended to frame men as part of the solution instead of part of the problem [42–44]. Further, the role of men in SRH is pivotal for their capacity to influence care-seeking (e.g., through financial control), to impact fertility decisions, and to provide proactive support to female partners. MAP for fertility care can be explored in settings such as The Gambia, with similar results than in other countries where this approach has targeted men’s involvement in SRH [45].

### Ensuring data-driven health policymaking

Measuring infertility is complicated due to the challenges in collecting data and the lack of appropriate indicators [46–48]. The lack of indicators may play a relevant role in the limited prioritization and implementation of fertility care programs [49]. In The Gambia, insufficient financial and logistical assistance, an inadequate referral system, insufficient funding for IT infrastructures, and scarce human resources all contribute to the operational challenges of the health management information system [50]. These challenges, coupled with the absence of routine data surveillance for infertility, do not help to advocate for international support, nor enable comparisons across countries [51]. The establishment and monitoring of these indicators would help Gambian policymakers in developing effective responses to address infertility and may support tailored interventions that meet greatly the health needs of the citizens. Although the data collected from fragile systems are often criticized as being over-explained, excluding them entirely diminishes the efforts to make changes because they can still expose the health concern in needs to be investigated, and act as tools for developing knowledge and setting priorities [52].

### Offering affordable IVF alternatives and regulations of standards

The Gambia has not yet introduced ART in its health system nor are any high-end medical techniques to assist infertile couples broadly available [18]. This offers a window of opportunity to the current health leadership as they are approaching the operationalization of fertility care. To this effect, considering that 48% of the population lives in poverty [53] and affording ART would be a difficult task for most Gambian couples, low-cost IVF initiatives may be explored. A few of those have been used by The Walking Egg (TWE) project and pioneered in African settings [54, 55]. In addition, to further reduce the cost of infertility care, the Gambian health leadership may explore partnerships with the private sector, facilitate transnational cooperation, and purchase ‘second-hand’ laboratory equipment to increase access to fertility care [56].

Whatever formula is adopted to cover or contribute to the cost of ART, such technologies must be medically and ethically regulated based on international guidelines, protocols, and evidence-based research. Regulations should be in place before the inclusion and implementation of ART in the health system. In fact, few studies cited that the regulatory component for assisted reproduction is often overlooked or missing, despite the provision of ART [57, 58]. Moreover, ART clinics usually adopt their own rules, which leads to different standards of care [59], contributes to inequality in access, and may facilitate professional liberty, with medical protocols not always in line with evidence-based standards or adapted to the needs of patients [60].

In The Gambia, considering that ART are not yet available, there is the opportunity to “start from scratch” with both the regulations and ART implemented at the same time with legislation, laws, and biomedical best practices. The establishment of a national fertility society and involvement with the African Federation of Fertility Societies (AFFS) may facilitate exchange and collaboration among society members, with the adoption of protocols and guidelines in alignment with those already existing in African countries that have included ART in their national policies [6, 10].

The Gambia might not be able to adopt, in the immediate future, comprehensive ART to assist its patients with infertility, but to comply with the UHC and strengthen its health system, it may ensure a minimum package of care for individuals and couples with fertility concerns.

### Improving knowledge of, and means for fertility care

Data triangulation highlighted how The Gambian health workforce needs fertility care training. Particularly, the medical education curricula need to be more comprehensive, not only focusing on the clinical practice but also providing mental and psychological support to couples with infertility [19, 60]. Furthermore, health staff providing infertility care need to learn how to manage patients’ expectations and any gaps in the adherence to treatments, because even in those settings where IVF is available, such as the case in many European countries, drop-out from infertility treatment is recorded as high [61] and this is strongly related to the mental and psychological burden of patients under fertility treatments [62–64].

### Specialized fertility care training

In terms of skills development for fertility care, in The Gambia, there are currently some initiatives for training on infertility management, mainly provided by the Merck Foundation through its program More than a Mother. This initiative supports the educational development of medical doctors in fertility, embryology, and sexual and reproductive medicine but is limited to this professional category, and other health providers, such as nurses and midwives, are currently excluded from the training. Considering that most of the secondary health facilities providing infertility services are managed by nurses and/or midwives, there is a substantial portion of the Gambian healthcare providers lacking specialized training for fertility care. Moreover, to the best of knowledge, no training or any other information on infertility prevention and awareness is provided to first-line healthcare providers, namely the Community Health Workers and the Community Birth Companions. The training of these workers is thought to be important due to the social stigma caused by infertility that often starts at household and community levels [65–67]. Moreover, these community workers can sensitize patients on the importance of early referral to initiate infertility screening and treatment as soon as possible [68].

### Enhancing collaboration among stakeholders and building links with the private sector

The triangulation of the datasets validated how the collaboration between the government and health facilities providing fertility care is somehow missing. This has resulted in an uneven distribution of infertility services among public health facilities, easing the rise of the private sector as the main provider of fertility care in the country, and exacerbating the restrictive access to, and the high cost of, infertility treatment [18, 69–71]. Further, the international development agencies currently in the country supporting multiple aspects of SRH have no targets to address infertility. This could be explained in several ways. First, (in)fertility care is not a priority on the agenda of international cooperation agencies. For this reason, the support of those agencies overlooks infertility and concentrates on addressing reproductive health matters considered to be of utmost importance [72]. Second, the private sector is in some way unregulated, with a census of the private clinics, including the health services provided and related costs, missing. Lastly, there are few mechanisms allowing the exchange of information between stakeholders, for example, thematic meetings or workgroups on infertility.

### Availability of infertility services in private care

Despite the unavailability of ART in The Gambia, the private sector largely provides the most comprehensive care for infertility but with services often not accessible to all people in need. This picture is not far from that in many other countries of SSA, where infertility services and particularly ART are mainly delivered by private clinics with little or no collaboration with the public health sector. In the few instances where fertility care is available in public hospitals (e.g., South Africa), this model requires large capital venture and investments in infrastructure and human resources [73]. Collaborative efforts in fertility care services, such as public-private partnership (PPP) may eventually reduce overhead costs through shared responsibilities [74] and could contribute in improving availability and access to services [75, 76].

#### Study limitations

*Informal health system*. The traditional medicine perspectives on fertility care were not captured in this study. However, the data triangulation showed the significant role of the informal health system in the management of infertility. Not involving the informal health system was a conscious decision to focus on the formal health sector because previous ethnographic research conducted in the country [39, 70, 77] provided some knowledge of the health-seeking processes taken by infertile women and men to solve their childlessness. However, an in-depth understanding of the herbalists’ and traditional doctors’ perspectives is needed.

*Service-users perspectives*. The research has explored only a one-sided perspective of the inclusion and implementation of fertility care, that of policymakers, policy implementers, and health practitioners, without any view from the service users, namely the patients.

#### Recommendations

*Male-factor infertility and participation in infertility management*. Men’s participation in the investigations and treatments for infertility are yet to be fully explored in The Gambia. There are multiple reasons why little attention has been dedicated to men with fertility issues but the most common are related to both sociocultural and gender biases impacting on the decision to seek help healthcare in case of diminished fertility. Evaluating men’s knowledge and awareness concerning fertility, and testing semen samples to identify factors and prevalence of male infertility is recommended as one of the next steps.

*Operational research*. Operational research can be applied both as a comparative study (how fertility care was implemented in a similar setting than The Gambia) as well as a monitoring and evaluation tool [78]. Operational research should include private care because much of what is currently provided in terms of fertility care is delivered by this sector.

*Educational curricula*. Emphasis on infertility should address the importance of having skilled health professionals appropriately trained in fertility care, also as fertility counselors. Currently, in The Gambia, fertility care matters are addressed as part of the medical and nursing high educational curricula but the degree of integration of infertility in these curricula is currently unknown. Measuring the impact of how medical academic training influences the management of infertility should be explored and could help in identifying grey areas in the educational curricula.

## Conclusions

The Gambian government has included fertility care in its new reproductive health strategic plan for the very first time. This inclusion came out from many years of engagement with policy actors, academic research, and grassroots activism targeting infertility and its harmful consequences. In the African health systems panorama, this engagement is relevant and may serve as an example for other countries in the region that aim to provide comprehensive management of infertility. The Gambian health system, however, will face some challenges in the implementation of fertility care, but those may be mitigated by maintaining a strong policy interest on infertility, involving Gambian men in sexual and reproductive health, collating data in a systematic way, and building durable collaborations with the private sector, and international partners to support the demand for and access to fertility care in the country.

## Supporting information

S1 Checklist Human participants research checklist. (DOCX)

S1 Table Triangulation protocol. (DOCX)
