# Supplementary material for: Policy action points and approaches to promote fertility care in The Gambia: Findings from a mixed-methods study
Source: PLoS One. 2024 May 14;19(5):e0301700. doi: 10.1371/journal.pone.0301700 (PMC11093356; doi:10.1371/journal.pone.0301700)
Supplement: S1 Table — (DOCX) [file pone.0301700.s002.docx]

**Supporting information 1: Triangulation Protocol**

| *Step* | *Activity* |
| --- | --- |
| - 1. Sorting | Findings from each dataset or method are classified into themes to determine areas of content overlap and/or divergence |
| - 1. Convergence coding matrix: | Identifying the themes from each dataset in order to determine the degree of convergence |
| - *Convergence* | Full agreement between the results from both datasets |
| - *Complementarity* | Findings from a dataset explain or complement data from the other dataset |
| - *Silence* | One dataset of findings covers the theme, whereas the other is silent on the same theme |
| - *Dissonance* | Disagreement between the results in both datasets |
| - 1. Convergence assessment | Reviewing the themes to provide a global assessment of the level of convergence. Document when and where datasets have different perspectives on convergence or dissonance of findings |
| - 1. Comparison | Compare the assessments of convergence or dissonance sorting from the united datasets to clarify the interpretation of results and determine the degree of triangulation Plan how to deal with differences of opinion and how to make final interpretation decisions |

*Table created by the author and adapted from (Farmer et al., 2006)*
